# Development and Validation of a Prediction Model for Perinatal Arterial Ischemic Stroke in Term Neonates

**DOI:** 10.1001/jamanetworkopen.2022.19203

**Published:** 2022-06-29

**Authors:** Ratika Srivastava, Mary Dunbar, Michael Shevell, Maryam Oskoui, Anna Basu, Michael John Rivkin, Eilon Shany, Linda S. de Vries, Deborah Dewey, Nicole Letourneau, Michael D. Hill, Adam Kirton

**Affiliations:** 1 Department of Pediatrics and Clinical Neurosciences, University of Calgary, Calgary, Alberta, Canada; 2 Department of Pediatrics, McGill University, Montreal, Quebec, Canada; 3 Department of Neurology/Neurosurgery, McGill University, Montreal, Quebec, Canada; 4 Newcastle upon Tyne Hospitals, National Health Service Foundation Trust, Newcastle upon Tyne, United Kingdom; 5 Department of Neurology, Boston Children’s Hospital, Boston, Massachusetts; 6 Department of Neurology, Harvard Medical School, Boston, Massachusetts; 7 Department of Neonatology, Soroka University Medical Center, Beer-Sheva, Israel; 8 Faculty of Health Sciences, Ben-Gurion University of the Negev, Beer-Sheva, Israel; 9 Department of Neonatology, University Medical Center Utrecht, Utrecht, the Netherlands; 10 Department of Pediatrics, Cumming School of Medicine, University of Calgary, Calgary, Alberta, Canada; 11 Department of Community Health Sciences, University of Calgary, Calgary, Alberta, Canada; 12 Owerko Centre at the Alberta Children’s Hospital Research Institute, Calgary, Alberta, Canada; 13 Hotchkiss Brain Institute, Cumming School of Medicine, University of Calgary, Calgary, Alberta, Canada; 14 Department of Pediatrics, Cumming School of Medicine, University of Calgary, Calgary, Alberta, Canada; 15 Department of Psychiatry, Cumming School of Medicine, University of Calgary, Calgary, Alberta, Canada; 16 Department of Community Health Sciences, Cumming School of Medicine, University of Calgary, Calgary, Alberta, Canada; 17 Department of Clinical Neurosciences, Cumming School of Medicine, University of Calgary, Calgary, Alberta, Canada; 18 Department of Community Health Sciences, Cumming School of Medicine, University of Calgary, Calgary, Alberta, Canada; 19 Department of Medicine, Cumming School of Medicine, University of Calgary, Calgary, Alberta, Canada; 20 Department of Radiology, Cumming School of Medicine, University of Calgary, Calgary, Alberta, Canada; 21 Hotchkiss Brain Institute, Cumming School of Medicine, University of Calgary, Calgary, Alberta, Canada; 22 Alberta Children’s Hospital Research Institute, University of Calgary, Calgary, Alberta, Canada

## Abstract

**Importance:**

Perinatal arterial ischemic stroke (PAIS) is a focal brain injury in term neonates that is identified postnatally but is presumed to occur near the time of birth. Many pregnancy, delivery, and fetal factors have been associated with PAIS, but early risk detection is lacking; thus, targeted treatment and prevention efforts are currently limited.

**Objective:**

To develop and validate a diagnostic risk prediction model that uses common clinical factors to predict the probability of PAIS in a term neonate.

**Design, Setting, and Participants:**

In this diagnostic study, a prediction model was developed using multivariable logistic regression with registry-based case data collected between January 2003, and March 2020, from the Alberta Perinatal Stroke Project, Canadian Cerebral Palsy Registry, International Pediatric Stroke Study, and Alberta Pregnancy Outcomes and Nutrition study. Criteria for inclusion were term birth and no underlying medical conditions associated with stroke diagnosis. Records with more than 20% missing data were excluded. Variable selection was based on peer-reviewed literature. Data were analyzed in September 2021.

**Exposures:**

Clinical pregnancy, delivery, and neonatal factors associated with PAIS as common data elements across the 4 registries.

**Main Outcomes and Measures:**

The primary outcome was the discriminative accuracy of the model predicting PAIS, measured by the concordance statistic (C statistic).

**Results:**

Of 2571 term neonates in the initial analysis (527 [20%] case and 2044 [80%] control individuals; gestational age range, 37-42 weeks), 1389 (54%) were male, with a greater proportion of males among cases compared with controls (318 [60%] vs 1071 [52%]). The final model was developed using 1924 neonates, including 321 cases (17%) and 1603 controls (83%), and 9 clinical factors associated with risk of PAIS in term neonates: maternal age, tobacco exposure, recreational drug exposure, preeclampsia, chorioamnionitis, intrapartum maternal fever, emergency cesarean delivery, low 5-minute Apgar score, and male sex. The model demonstrated good discrimination between cases and controls (C statistic, 0.73; 95% CI, 0.69-0.76) and good model fit (Hosmer-Lemeshow *P* = .20). Internal validation techniques yielded similar C statistics (0.73 [95% CI, 0.69-0.77] with bootstrap resampling, 10-fold cross-validated area under the curve, 0.72 [bootstrap bias–corrected 95% CI, 0.69-0.76]), as did a sensitivity analysis using cases and controls from Alberta, Canada, only (C statistic, 0.71; 95% CI, 0.65-0.77).

**Conclusions and Relevance:**

The findings suggest that clinical variables can be used to develop and internally validate a model to predict the risk of PAIS in term neonates, with good predictive performance and strong internal validity. Identifying neonates with a high probability of PAIS who could then be screened for early diagnosis and treatment may be associated with reductions in lifelong morbidity for affected individuals and their families.

## Key Points

**Question:**

Can common clinical factors be used to develop and internally validate a risk prediction model for perinatal arterial ischemic stroke (PAIS) in term neonates?

**Findings:**

In this diagnostic study of 2571 term neonates, a prediction model for risk of PAIS that included 1924 neonates and 9 clinical factors (maternal age, tobacco exposure, recreational drug exposure, preeclampsia, chorioamnionitis, intrapartum maternal fever, emergency cesarean delivery, low 5-minute Apgar score, and male sex) had good discrimination and model fit between case and control individuals.

**Meaning:**

Prediction models for PAIS may help identify neonates at risk of PAIS who should be screened for early diagnosis and intervention.

## Teaser

This diagnostic study assesses the accuracy and internal validity of a prediction model developed to estimate the risk of perinatal arterial ischemic stroke in term neonates using clinical pregnancy, delivery, and fetal factors.

## Introduction

Perinatal stroke is a focal vascular brain injury defined as occurring from the fetal period to 28 days of postnatal life.^1^ With an overall incidence of up to 1 in 1000 live term births, the most focused lifetime risk for stroke occurs near birth,^2^ and perinatal stroke is the primary cause of hemiparetic cerebral palsy.^3^ The most common type of perinatal stroke is perinatal arterial ischemic stroke (PAIS), classified further by timing of presentation. PAIS is most often diagnosed as neonatal AIS in term neonates, which manifests acutely as seizures or encephalopathy.^4^ PAIS may also go undetected in the neonatal period and present in late infancy or early childhood, when it is termed *arterial presumed perinatal ischemic stroke*.^5^ Both are considered within a spectrum of the same disease.

The timing of perinatal stroke allows for unique pathophysiological considerations, including biological factors between the mother and fetus and peripartum-specific factors, such as the placenta, labor and delivery, fetal transition, and adaptive alterations of the coagulation system in both mother and neonate.^4,6^ PAIS may be associated with an underlying condition such as complex congenital heart disease^7^ or bacterial meningitis,^8^ although additional contributing factors may still be present. Studies of PAIS have suggested associations with maternal and pregnancy factors, such as nulliparity, preeclampsia, and gestational diabetes^9,10,11^; intrapartum factors, such as maternal fever and chorioamnionitis^9,11,12,13^; and fetal or neonatal factors, such as fetal heart rate abnormalities, intrauterine growth restriction, meconium staining, and male sex.^14,15,16^ These findings, however, have been inconsistent and were likely affected by variable terminologies and modest sample sizes. Without a way to identify neonates at risk of PAIS who appear to be healthy, early diagnosis of PAIS and strategies for prevention are challenging.

Placental pathology is suspected to be a common factor associated with PAIS.^17^ The placenta provides oxygenation and nutrition to the fetus and is a direct source of thromboembolism to the brain via the fetal circulation, which lacks the thrombus-filtering capacity of the postnatal pulmonary circulation. Although histopathology is challenging to obtain, perinatal stroke has been associated with a variety of placental conditions^18,19^ through mechanisms of maternal or fetal vascular malperfusion, thromboinflammatory processes, and infection.^20,21,22^ Clinical factors, such as the frequent bilaterality of lesions in PAIS (implying a proximal embolic source^23^) and the low recurrence risk (<1%) of PAIS,^24,25^ are also consistent with a primary placental mechanism.

Prediction models have proved to be valuable in the primary prevention of adult stroke^26^ and have shown patient factors, such as history of diabetes, hypertension, and atrial fibrillation, to be candidate predictors.^27^ To our knowledge, such models have not yet been developed or validated for perinatal stroke because the complex and unmeasurable nature of PAIS pathophysiology makes primary prevention in utero a challenge. Prediction and early diagnosis of PAIS could allow close monitoring in the perinatal period, with possible implications for emerging acute treatments^28^ and early rehabilitation^29,30,31^ to optimize outcomes.

Using a large, well-characterized sample of PAIS cases, we sought to develop and validate a diagnostic risk-prediction model based on common clinical perinatal factors that estimates the probability of PAIS in a term neonate. A secondary objective was to explore whether the factors identified a priori in predicting PAIS could support a placental mechanism.

## Methods

### Population and Data Sources

In this diagnostic study, PAIS cases were collected from 3 sources: the Alberta Perinatal Stroke Project, the Canadian Cerebral Palsy Registry, and the International Pediatric Stroke Study. The Alberta Perinatal Stroke Project, established in 2008, is a research cohort with prospective (2008-2017) and retrospective (1990-2008) enrollment at a single tertiary care pediatric center (Alberta Children’s Hospital) in Alberta, Canada.^32^ The Canadian Cerebral Palsy Registry, established in 2003, is a multiregional prospective Canadian registry of children with cerebral palsy.^33^ A system for confirming and classifying perinatal stroke (arterial and venous) in participants with hemiparetic cerebral palsy was recently validated in this registry.^34^ The International Pediatric Stroke Study, established in 2006, is a clinical research registry of pediatric stroke that stores medical and imaging data for international collaborative research.^23^ These data are collected using standardized procedures from International Pediatric Stroke Study investigators in more than 15 countries, with approximately 70% of participants residing in Canada and the US.^23^ Case data for the current study were collected from January 2003 to March 2020, with data analysis completed in September 2021. Because these 3 registries include overlapping catchment areas, source data were cross-referenced to ensure no participants were included more than once. The University of Calgary and Alberta Health Services research ethics boards approved the study and waived informed consent owing to the retrospective study design and analysis. This study followed the Transparent Reporting of a Multivariable Prediction Model for Individual Prognosis or Diagnosis (TRIPOD) reporting guideline.^35^

Data for healthy control individuals were obtained from the Alberta Pregnancy Outcomes and Nutrition study,^36^ established in 2009 as a population-based prospective cohort of pregnant women in Alberta. This longitudinal cohort study collected pregnancy, delivery, and neonatal data and has followed child health outcomes to 12 years of age. The Alberta Pregnancy Outcomes and Nutrition common data elements have previously been used as control variables in studies of factors associated with PAIS.^34^

### Criteria for Inclusion and Exclusion

Registries were reviewed for eligible case and control individuals. Participants were selected based on the following criteria: (1) birth between the year of registry establishment and March 2020, (2) term birth (≥37 weeks’ gestation), and (3) no medical comorbidities associated with a stroke diagnosis (eg, meningitis, major congenital anomaly). Stroke case definitions were applied across the 3 case registries (Alberta Perinatal Stroke Project, Canadian Cerebral Palsy Registry, and International Pediatric Stroke Study) and included a magnetic resonance imaging–confirmed diagnosis of AIS presumed to have occurred in the neonatal period as established by clinical-radiographic diagnostic criteria.^5,23^ Healthy controls required normal motor development at 3 years of age to rule out delayed presentation of stroke. Participants were excluded if caregiver consent was incomplete or if more than 20% of data fields were missing.

### Common Data Elements as Predictor Variables

The 4 source registries collected comparable common data elements that have been associated with perinatal stroke pathogenesis and presentation. Study variables were identified from these common data elements and included maternal, pregnancy, obstetric, fetal, and neonatal factors. To ensure consistency across the perinatal literature, medical definitions of these variables have been stated in the National Institute of Neurological Disorders and Stroke, National Institutes of Health, Common Data Elements.^37^ Variables were only included in this study if they were consistently defined and directly comparable across the source registries according to their data codebooks, with any uncertainty resolved with each study coordinator to ensure accuracy. Data for certain variables were recoded from ordinal or continuous scales into binary measures to ensure that the variable’s presence was captured consistently in the prediction model (eTable in the Supplement).

### Statistical Analyses

#### Model Specification

Descriptive statistics were used to evaluate data for completeness and to identify the prevalence of each study variable among cases and controls. Candidate predictors for the model were identified a priori based on peer-reviewed research literature^10,13,14,15,16,17,18,19,20,21,22,34,38,39,40,41,42^ that previously showed them to be associated with PAIS and/or to have biological plausibility in placental pathology. Thus, the selection of candidate predictors was based on known associations (higher pretest probability) rather than prompted by the current data.

#### Model Development and Validation

Univariable analyses using logistic regression were done to explore individual associations between each selected clinical variable and the outcome of PAIS. A diagnostic prediction model was then developed using multivariable logistic regression of main effects of these candidate variables to predict the probability of PAIS. Complete case analysis was used to address missing data, whereby only participants for whom there were no missing data for the candidate predictors were included in the model. The primary outcome was discriminative accuracy of the model in predicting PAIS, measured by the concordance statistic (C statistic), shown as the area under the receiver operating characteristic curve. The C statistic is the probability that a randomly selected individual who experienced the outcome (ie, PAIS) would have a higher predicted probability of having the outcome occur than would a randomly selected healthy control. Values greater than 0.7 indicate good model discrimination.^43^ Model fit was assessed using the Hosmer-Lemeshow test. Internal validation was done using bootstrap resampling and 10-fold cross-validation. As a sensitivity analysis, the model was applied to only cases and controls from Alberta, Canada, and a C statistic representing a single, local population was obtained. All available data were used for model development, with resampling methods used for internal validation.^44^ Analyses were conducted using Stata, version 16 (StataCorp LLC). Two-sided *P* = .05 was considered significant. The final model was presented as a regression formula using the candidate predictors weighted by their coefficients to estimate a term neonate’s predicted risk of PAIS.

## Results

### Baseline Characteristics of Study Participants

**Figure 1.**
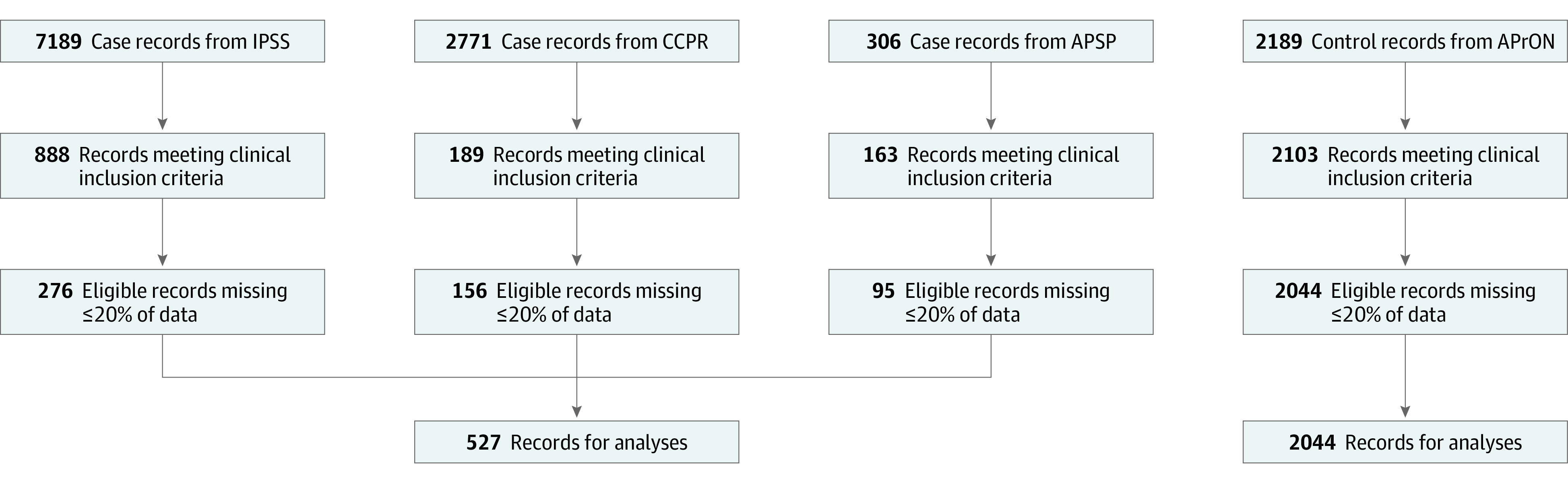
Selection of Case and Control Records Eligible records were identified based on the following clinical inclusion criteria: term neonate, no underlying comorbidities, and case individuals with perinatal arterial ischemic stroke or control individuals with normal development until 3 years of age. Records with 20% or more missing data were excluded. APrON indicates Alberta Pregnancy Outcomes and Nutrition; APSP, Alberta Perinatal Stroke Project; CCPR, Canadian Cerebral Palsy Registry; and IPSS, International Pediatric Stroke Study.

A total of 2571 participants were included in the initial analysis; 527 (20%) were case patients with PAIS from the 3 case registries and 2044 (80%) were healthy controls (Figure 1). Clinical characteristics are shown in Table 1. Of the 2571 participants, 1389 (54%) were male, with a greater proportion of males in the case group compared with the control group (318 [60%] vs 1071 [52%]). All participants were term neonates born between 37 and 42 weeks’ gestation.

**Table 1.** Clinical Characteristics of Case Patients With Perinatal Arterial Ischemic Stroke and Control Individuals

Variable	Controls (n = 2044)		Cases (n = 527)		Total No. (%) (N = 2571)
	No. (%)	Missing data, %	No. (%)	Missing data, %	
Maternal age, mean (SD), y	32 (4)	15	30 (5)	11	31 (5)
Primigravida	857 (42)	0	116 (24)	23	973 (38)
Tobacco exposure in pregnancy	117 (6)	0	47 (12)	24	164 (7)
Alcohol use in pregnancy	174 (9)	7	25 (6)	25	199 (9)
Recreational drug exposure in pregnancy	14 (1)	7	20 (5)	24	34 (1)
Gestational diabetes	37 (7)	0	75 (4)	3	112 (4)
Gestational hypertension	126 (6)	0	37 (7)	0	177 (7)
Preeclampsia	17 (1)	0	16 (3)	3	33 (1)
Clinical chorioamnionitis	8 (<0.1)	0	22 (4)	0	30 (1)
Placenta previa	7 (<0.1)	0	6 (1)	0	13 (1)
Meconium	404 (20)	1	106 (31)	35	510 (22)
Maternal fever intrapartum	78 (4)	0	49 (10)	3	127 (5)
Vaginal delivery	1527 (75)	0	286 (54)	0	1813 (71)
Cesarean delivery		0		0	
Planned	256 (12)	0	80 (15)	0	336 (13)
Emergency	257 (13)	0	155 (30)	0	412 (16)
Placental abruption	9 (<0.1)	0	2 (<0.1)	0	11 (<0.1)
Apgar score, mean (SD)		0		0	
At 1 min	8 (2)	0	7 (3)	14	8 (2)
At 5 min	9 (1)	0	8 (2)	12	9 (1)
Resuscitation required	972 (48)	0	184 (37)	5	1156 (45)
Neonate sex					
Female	973 (48)	0	209 (40)	0	1182 (46)
Male	1071 (52)	0	318 (60)	0	1389 (54)
Head circumference, mean (SD), cm	35 (2)	8	35 (2)	42	35 (2)

Tobacco and recreational drug (substance) use in pregnancy were present in increased proportions in the case cohort, as were certain intrapartum factors, such as chorioamnionitis and maternal fever. Clinical chorioamnionitis was present in 22 PAIS cases (4%) and 8 controls (<1%). With regard to mode of delivery, a higher proportion of controls was delivered vaginally (1527 [75%] vs 286 [54%]), whereas a higher proportion of cases was delivered via emergency caesarian delivery (155 [30%] vs 257 [13%]). The percentage of missing data for each clinical factor, where applicable, is also shown in Table 1.

### Model Specification

Preliminary univariable analyses were done to show possible associations between each individual predictor and outcome (Table 2). Of the 18 study variables identified in Table 2, the 9 following factors were selected as candidate predictors for the model because existing literature and biological plausibility with regard to the placental hypothesis of PAIS supported that these factors were associated with a higher pretest probability of PAIS (Table 3)^9,10,13,14,15,16,17,18,20,21,22,34,38,39,40,41,42,45,46^: maternal age, tobacco exposure in pregnancy, recreational drug (substance) exposure in pregnancy, preeclampsia, chorioamnionitis, maternal fever intrapartum, emergency cesarean delivery, low Apgar score (<7) at 5 minutes, and male sex. All 9 variables selected as candidate predictors were significantly associated with the outcome in univariable analyses, and no further variable selection was undertaken; all 9 variables were included in the final model.

**Table 2.** Univariable Associations Between Predictor Variables and Perinatal Arterial Ischemic Stroke

Variable	Odds ratio (95% CI)
Maternal age	0.91 (0.89-0.93)
Tobacco exposure in pregnancy	2.01 (1.38-2.91)
Alcohol use in pregnancy	0.67 (0.41-1.03)
Recreational drug use in pregnancy	7.09 (3.37-15.30)
Gestational diabetes	2.04 (1.32-3.11)
Gestational hypertension	1.63 (1.13-2.31)
Preeclampsia	3.77 (1.77-7.98)
Placenta previa	3.35 (0.92-11.68)
Chorioamnionitis	11.09 (4.71-28.93)
Maternal fever intrapartum	2.57 (1.86-4.04)
Vaginal delivery	0.41 (0.33-0.50)
Cesarean delivery	
Planned	1.26 (0.94-1.66)
Emergency	2.91 (2.30-3.68)
Placental abruption	0.86 (0.09-4.18)
Apgar score	
At 1 min	0.72 (0.69-0.76)
At 5 min	0.59 (0.53-0.65)
Resuscitation required	0.63 (0.52-0.78)
Male sex	1.38 (1.13-1.69)

**Table 3.** Candidate Predictors Independently Associated With PAIS in Multivariable Logistic Regression

Variable	Rationale for selection	OR (95% CI)
Pregnancy factors		
Maternal age	Previous association with PAIS^34^ and placental disease (ie, fetal vascular malperfusion)^39^	0.91 (0.89-0.93)
Tobacco exposure in pregnancy	Previous association with PAIS^10,34^ and placental disease^38^	1.23 (1.00-2.57)
Recreational drug exposure in pregnancy	Previous association with PAIS^10,34^ and placental vascular malperfusion^45^	5.66 (2.45-13.09)
Preeclampsia	Previous association with PAIS^13,34^ and placental disease (maternal and fetal vascular malperfusion)^21,40^	2.36 (0.99-5.58)
Labor and delivery factors		
Chorioamnionitis	Previous association with PAIS^13,18,22,34,41^ and placental inflammatory/thromboembolic processes^20^	3.63 (1.31-10.03)
Maternal fever intrapartum	Previous association with PAIS^13,34^ and placental inflammatory/thromboembolic processes^18,42^	1.68 (1.03-2.75)
Emergency cesarean delivery	Previous association with PAIS^13,34,41^ and difficult transition to extrauterine life^17,21^	1.65 (1.19-2.27)
Neonatal factors		
Low Apgar score (<7) at 5 min	Previous association with PAIS^9,13,34,41^ and difficult transition to extrauterine life^20,46^	5.40 (3.50-8.33)
Male sex	Previous association with PAIS^13,14,15,16,41^	1.33 (1.02-1.73)

Abbreviation: PAIS, perinatal arterial ischemic stroke.

Table 3 shows the multivariable regression output on which the prediction model was based and the rationale for including each variable in the model. Recreational substance exposure was associated with increased odds of PAIS by 5.66 times (OR, 5.66; 95% CI, 2.45-13.09), and tobacco exposure was not found to have a significant association in adjusted analysis (OR, 1.23; 95% CI, 1.00-2.57). The presence of chorioamnionitis was associated with increased odds of PAIS by 3.63 times (OR, 3.63; 95% CI, 1.31-10.03). A low 5-minute Apgar score was associated with increased odds of PAIS by 5.4 times (OR, 5.40; 95% CI, 3.50-8.33). In addition, the odds of PAIS were slightly higher among male neonates than among female neonates (OR, 1.33; 95% CI, 1.02-1.73).

### Model Development and Validation

**Figure 2.**
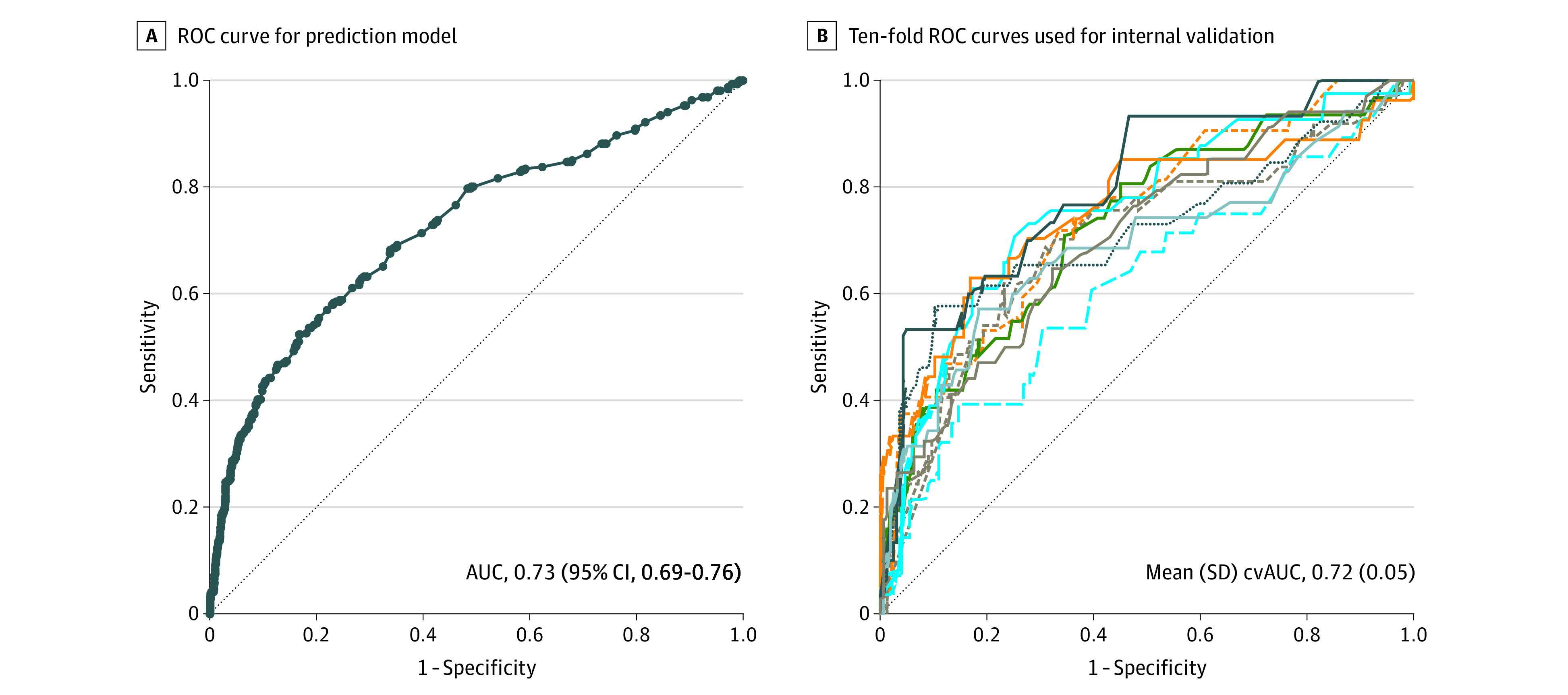
Results of Perinatal Arterial Ischemic Stroke Risk Prediction Model Validation Analysis B, Each colored line represents 1 of the receiver operating characteristic (ROC) curves for the results in the 10-fold cross-validation. Dashed diagonal lines indicate no predictive value. AUC indicates area under the receiver operating characteristic curve; cv, cross-validated.

The final risk-prediction model was developed using 1924 participants, including 321 cases (17%) and 1603 controls (83%). Model performance measures, presented in Figure 2, demonstrated good discrimination between cases and controls (C statistic, 0.73; 95% CI, 0.69-0.76; intercept, –2.65; slope, 5.43) (Figure 2A) and model fit (Hosmer-Lemeshow *P* = .20). Sensitivity, or detection rate, was 11% with a false-positive rate of 1%. There was also indication of overestimation (calibration intercept <0) without overfitting (calibration slope >1). Two methods of internal validation, bootstrap resampling and k-fold cross-validation, were used and yielded C statistics similar to that in the original model. Bootstrapped results showed a C statistic of 0.73 (95% CI, 0.69-.077), and the mean 10-fold cross-validated area under the curve was 0.72 (bootstrap bias–corrected 95% CI, 0.69-0.76) (Figure 2B).

A sensitivity analysis was done using only Alberta Perinatal Stroke Project case data and a randomly selected subset of Alberta Pregnancy Outcomes and Nutrition controls because participants in both groups were from the same Alberta population and thus were presumed to be the most homogeneous. This model included 479 participants (87 cases [18%] and 392 controls [82%]) and demonstrated a C statistic of 0.71 (95% CI, 0.65-0.77) and good model fit (Hosmer-Lemeshow *P* = .86).

The final model developed to estimate the individual predicted risk of PAIS in a term neonate was presented as a regression formula:

predicted risk of PAIS in a term neonate = (1/1 + e^–^*^t^*), where

*t* = 1.26 + (−0.1 ×  maternal age in years) + (0.21 × tobacco exposure) + (1.73 × substance exposure) + (0.86 × preeclampsia) + (1.29 × chorioamnionitis) + (0.52 × maternal fever intrapartum) + (0.50 × emergency cesarean delivery) + (1.69 × low 5-minute Apgar score) + (0.28 × male neonate), substituting 1 for present and 0 for absent for each variable (excluding maternal age in years).

## Discussion

In this diagnostic study, we developed an internally valid, diagnostic clinical prediction model to quantify the risk of PAIS in term neonates. Using data from 4 study cohorts of women and infants and 9 commonly available clinical factors, this model showed good predictive performance (C statistic, 0.73; 95% CI, 0.69-0.76) and strong internal validity. Similar findings were obtained using a subset of the Alberta data only (C statistic, 0.71; 95% CI, 0.65-0.77). The results suggest that simple clinical prediction models may improve estimations of the risk of PAIS occurrence by 20-fold compared with current birth prevalence rates.^2^ Biologically, these data provide indirect support for the placental embolism hypothesis for PAIS pathogenesis.

Although data on direct examination of the placenta were not available in this large-scale study, key clinical factors associated with abnormal placental physiology and histology in PAIS^18,19,21^ were used as surrogates to consider the role of placental dysfunction. Chorioamnionitis has the most direct association with placental disease,^47^ and in this study, when chorioamnionitis was present, the odds of PAIS were increased by a factor of 3.63 (95% CI, 1.31-10.03). The wide 95% CI, however, suggests a low prevalence of this predictor in our data set; it was present in only 30 neonates (1%) in the study population. Of importance, the proportion of chorioamnionitis in the control group (<1% [8 individuals]) was similar to the known North American population-based prevalence,^47^ whereas the proportion in the case group was 4% (22 individuals), supporting the importance of this factor in PAIS. This finding also supports well-established associations between chorioamnionitis and perinatal brain injury in term neonates.^9,20^

Recreational drug (substance) exposure in pregnancy was significantly associated with increased odds of PAIS (OR, 5.66; 95% CI, 2.45-13.09), whereas tobacco exposure was not (OR, 1.23; 95% CI, 1.00-2.57). Although the effects of tobacco on the placenta are presumed to occur through chronic reduction of blood flow that creates a pathologically hypoxic environment,^38^ vasoactive drugs such as cocaine or methamphetamine may be more likely to prompt a thromboembolic process in the placenta,^45^ resulting in a focal arterial ischemic injury in the fetal or neonatal brain. Given the power of the sample in our study and that rates of both recreational substance and tobacco use among controls approximated published rates,^36^ our findings suggest that these potentially modifiable factors associated with PAIS require further investigation.

Prediction models provide diagnostic probabilities and potentially impact clinical practice when actions can be taken with regard to the prediction. With the inclusion of neonatal clinical factors, some of which can only be collected immediately after birth, the goal of this study’s prediction model was to identify neonates at risk for PAIS for early diagnosis and treatment to prevent secondary complications. Although neuroimaging such as magnetic resonance imaging is required to confirm a PAIS diagnosis, this technique is not always available or suitable for a neonate who is clinically unstable. Delay of a PAIS diagnosis by days, months, or even years is common in the case of presumed perinatal stroke.^48^ An effective prediction model could help determine which neonates should receive a screening evaluation, such as a cranial ultrasonography, a noninvasive and inexpensive bedside test, if risk of perinatal stroke was found to be present.^49^ Acute treatments for PAIS, such as stem cell therapy and erythropoietin, are currently being studied,^28,50^ and if PAIS is highly suspected in a neonate, earlier diagnosis leading to treatment may optimize outcomes.^51^ In addition, this study’s model may be particularly useful to identify seemingly asymptomatic neonates with perinatal stroke (ie, presumed PAIS), because their window for neuroprotection and early therapy is wider and often missed. Unique inflammatory biomarkers collected from acute blood samples from neonates have also been associated with PAIS.^52^ Application of our clinical prediction model could be used in combination with such biomarkers within the first 48 hours of life to further enhance early identification.

The next step for this diagnostic clinical prediction model of PAIS would be to assess its parameters at various predicted thresholds to better establish risk groups for optimal sensitivity. The complexity of these data lends itself to higher-level analyses, and efforts are under way to use machine learning techniques to make more accurate data-driven predictions. This study may expand the way clinicians and researchers think about perinatal stroke and stroke prediction and prevention and will hopefully serve as a foundation on which future research can be based.

### Strengths and Limitations

This study has strengths. The sample size was more than 500 PAIS cases, and thus, to our knowledge, the study included the largest case group among existing case-control studies. The unique study methods identified easily measured and well-defined clinical factors as predictors for PAIS. In addition, our approach of using existing literature to define relevant factors and then validating them collectively using this data set supports the consistency in the literature on key variables associated with PAIS. Perinatal stroke is a rare disease, and randomized clinical trials are not possible because many variables (eg, emergency cesarean delivery, chorioamnionitis) and the outcome of perinatal stroke itself cannot be randomly assigned or manipulated; therefore, we believe that analyses such as ours may be the best way to develop predictive models for early PAIS detection.

This study also has limitations. There was a lack of external validation, which was not feasible with the available data. Certain factors known to be associated with perinatal stroke, such as nulliparity, meconium, and abnormal fetal heart rate, were not consistently captured across the source registries, and thus, the association of these potential predictors with PAIS could not be included in the model. In addition, although PAIS cases were obtained from local, national, and international registries to increase study power, controls were obtained only from the Alberta population and may not have had the same baseline rates of certain factors (ie, tobacco exposure) as the US population^53^ or other populations worldwide. The sensitivity analysis using only cases and controls from Alberta attempted to mitigate some of this imbalance and revealed similar results.

## Conclusions

This diagnostic study showed that clinical variables may have predictive utility in identification of neonates at risk of PAIS. Clinicians often rely on their judgement and limited experiences in predicting the likelihood of PAIS, which is challenging for nonexperts when assessing a rare disease of poorly understood pathogenesis. To date, validated guidelines to aid in such prediction do not exist; however, the clinical variables included in this study are readily available and intuitively considered when making an informed determination of risk in neonates with neurological concerns. The process of updating a clinician’s prior beliefs about whether an individual has PAIS is inherently bayesian,^54^ and these intuitions might be supported by the addition of a risk prediction model that provides rationale for a higher or lower clinical index of suspicion. Because the prevalence of acutely symptomatic PAIS is less than 1 in 2500 live term births^2^ and the clinical recognition rate is also low, this model’s detection rate of 11% may substantially improve the identification of cases. In an era of precision medicine, identifying key factors associated with PAIS may have marked clinical impact in reducing the burden of perinatal stroke on patients and families.

## Notes

Supplement. **eTable.** Full List of Variables Collected Across Source Registries and Their Coding for the Present Study
