# Supplementary material for: Development and Validation of a Prediction Model for Perinatal Arterial Ischemic Stroke in Term Neonates
Source: JAMA Netw Open. 2022 Jun 29;5(6):e2219203. doi: 10.1001/jamanetworkopen.2022.19203 (PMC9244611; doi:10.1001/jamanetworkopen.2022.19203)
Supplement: Supplement. — eTable. Full List of Variables Collected Across Source Registries and Their Coding for the Present Study [file jamanetwopen-e2219203-s001.pdf]

Supplementary Online Content

Srivastava R, Dunbar M, Shevell M, et al. Development and validation of a prediction model for perinatal arterial ischemic stroke in term neonates. *JAMA Netw Open*. 2022;5(6):e2219203. doi:10.1001/jamanetworkopen.2022.19203

eTable. Full List of Variables Collected Across Source Registries and Their Coding for the Present Study

This supplementary material has been provided by the authors to give readers additional information about their work.

© 2022 Srivastava R et al. *JAMA Network Open*.

eTable. Full list of variables collected across source registries and their coding for the present study

Category	Variable name	Variable description	Variable type in current study	Source registry coding			
				IPSS	APSP	CCPR	APRoN
Demographic	PatientID	Anonymized ID number	N/A				
Demographic	source	Registry source	Nominal (0=APRON; 1=APSP; 2=CCPR; IPSS=3)				
Pregnancy	matage	Maternal age	Continuous, years	continuous	continuous	continuous	continuous
Pregnancy	primigravida*	First pregnancy	Binary (0=no; 1=yes)	count (gravida)	count (gravida)	count (total prior preg)	count
Pregnancy	miscarri*	Previous of miscarriages <20 weeks GA	Binary (0=no; 1=yes)	count	count	count	count
Pregnancy	infertility	History of infertility	Binary (0=no; 1=yes)	yes/no	yes/no	yes/no	yes/no
Pregnancy	tobac*	Tobacco use in pregnancy	Binary (0=no; 1=yes)	yes/no	ordinal (range # cigarettes/week)	yes/no	count (frequency)
Pregnancy	alcohol*	Alcohol use in pregnancy	Binary (0=no; 1=yes)	yes/no	ordinal (serving/week)	yes/no	count (frequency)
Pregnancy	substance*	Recreational drug use in pregnancy	Binary (0=no; 1=yes)	yes/no	yes/no	yes/no	count (frequency)
Pregnancy	diabgest	Gestational diabetes	Binary (0=no; 1=yes)	yes/no	yes/no	yes/no	yes/no
Pregnancy	htngest	Gestational hypertension	Binary (0=no; 1=yes)	yes/no	yes/no	yes/no	yes/no
Pregnancy	preeclamp	Pre-eclampsia	Binary (0=no; 1=yes)	yes/no	yes/no	yes/no	yes/no
Pregnancy	previa	Placenta previa	Binary (0=no; 1=yes)	yes/no	yes/no	yes/no	yes/no
Delivery	chorio	Chorioamnionitis	Binary (0=no; 1=yes)	yes/no	yes/no	yes/no	free text
Delivery	prolongrup	Prolonged rupture of membranes	Binary (0=no; 1=yes)	yes/no	yes/no	yes/no	yes/no
Delivery	mec*	meconium	Binary (0=no; 1=yes)	yes/no	ordinal (thickness)	Not captured	ordinal (thickness)
Delivery	matfev	Maternal fever intrapartum	Binary (0=no; 1=yes)	yes/no	yes/no	yes/no	yes/no
Delivery	del_mode*	Mode of delivery	Binary (0=C-section; 1=vaginal)	nominal	nominal	nominal	yes/no
Delivery	abrupt	Placental abruption	Binary (0=no; 1=yes)	nominal	yes/no	nominal	free text
Neonatal	apgar1	Apgar score at 1 minute	Ordinal (0-10 scale)	ordinal	ordinal	ordinal	ordinal
Neonatal	apgar5	Apgar score at 5 minutes	Ordinal (0-10 scale)	ordinal	ordinal	ordinal	ordinal
Neonatal	resus*	Resuscitation required	Binary (0=no; 1=yes)	yes/no	nominal	yes/no	nominal
Neonatal	sex	Neonate sex	Binary (0=female; 1=male)	binary	binary	binary	binary
Neonatal	gestage*	Gestational age	Binary (0=37-40+6 weeks; 1=41+ weeks)	continuous (wks/days)	ordinal (grouped weeks)	continuous (weeks/days)	continuous (weeks/days)
Neonatal	bweight	Birth weight	Continuous, grams	continuous	continuous	continuous	continuous
Neonatal	hc	Head circumference	Continuous, centimetres	continuous	continuous	continuous	continuous
Outcome	presentation*	No stroke, NAIS, or APPIS	Nominal (0=control; 1=NAIS; 2=APPIS)	nominal	nominal	inferred from clinical factors**	nominal
**Outcome**	**stroke**	**Control or case**	**Binary (0=control, no stroke; 1=case, stroke)**	nominal	nominal	nominal	nominal

*indicates recoding due to variations in source registry format

**neonatal seizure/encephalopathy used as surrogate leading to NAIS presentation

© 2022 Srivastava R et al. *JAMA Network Open*.
